# Evaluating the necessity of bone augmentation for distal radius fracture fixed with a volar locking plate: a retrospective study

**DOI:** 10.1186/s12891-020-03203-7

**Published:** 2020-03-19

**Authors:** Feng-Shuo Chang, Chih-Hui Chen, Cheng-Hung Lee, Kun-Tsan Lee, Yi-Cheng Cho

**Affiliations:** 1 Department of Orthopedics, Taichung Veterans General Hospital, 1650, Taiwan Boulevard Sect. 4, Taichung, 40705 Taiwan; 2 School of Medicine, National Yang-Ming University, No.155, Sec. 2, Li-Nong Street, Pei-Tou District, Taipei City 112, Taiwan; 3 Department of Food Science and Technology, Hung Kuang University, Taichung, Taiwan; 4 Department of Veterinary Medicine, College of Veterinary Medicine, National Chung-Hsing University, Taichung, Taiwan

**Keywords:** Volar locking plate, Distal radius fracture, Bone augmentation, Radiographic outcomes, Internal fixation

## Abstract

**Background:**

Multiple approaches for fixation of distal radius fractures exist; nonetheless, there is no consensus on the optimal treatment for these injuries. Although using volar locking plates has become increasingly common as a surgical intervention, the usefulness of bone augmentation remains debatable. Therefore, this study aimed to evaluate the necessity of bone augmentation for distal radius fractures fixed with a volar locking plate.

**Methods:**

This retrospective study enrolled patients with a single distal radius fracture treated with a volar locking plate between January 2014 and December 2016. Overall, 105 fractures were included and divided into two groups (non-bone augmentation: group 1, *n* = 88; bone augmentation: group 2, *n* = 17). Images were reviewed, and dorsal cortex collapse, volar tilting, and radial height and inclination were measured immediately after surgery and at the 6-month follow-up.

**Results:**

Both groups exhibited significant differences in dorsal collapse (*p* < 0.001 and *p* = 0.001, respectively) and radial height shortening (*p* < 0.001 and *p* = 0.039, respectively); volar tilting and radial inclination did not differ significantly. There was no difference in the degree of dorsal collapse (*p* = 0.715) and radial height shortening (*p* = 0.651) between the two groups. Of the 105 fractures, 54 were identified as comminuted type according to the AO classification (A3, C2, and C3), and similar radiographic outcomes were noted.

**Conclusions:**

Volar locking plates for the treatment of distal radius fractures with or without bone augmentation do not affect the radiographic outcomes. In comminuted fractures, additional bone augmentation is unnecessary if intraoperative anatomical reduction and fixation are performed when possible.

## Background

Distal radius fracture is one of the most common bone fractures [1]. A previous study indicated that the average cost of care for a distal radius fracture patient was up to 8 thousand dollars in the United States during 2009–2015 [2]. Many treatment approaches, including nonsurgical (closed reduction and casting) and surgical (percutaneous fixation, external fixation, and open reduction and internal fixation) techniques for distal radius fractures have been established, and maximal restoration of anatomical alignment and early motion of the wrist is desirable [3–5].

The volar locking plate became commercially available in 2000. Its design allows better fixation of osteoporotic and comminuted bone fractures that were previously difficult to manage. A proper volar approach can help achieve intraoperative anatomical reduction, while using a low-profile locking plate can reduce compression and irritation of the median nerve and flexor tendons. Due to these advantages, the volar locking plate is increasingly used for surgical fixation and has proven effective [6–9].

In terms of surgical treatment, bone augmentation has been performed using a bone graft or bone substitute to fill metaphyseal bone defects caused by fracture, as it is believed to prevent fracture collapse and promote bone healing, especially in osteoporotic and comminuted bone fractures. However, to the best of our knowledge, there is currently no strong evidence that suggests the efficacy of bone augmentation and the indications for its use. Furthermore, bone augmentation is also associated with risks or complications, such as inflammation or improper healing [10–12].

For osteoporotic and comminuted bone fractures, the use of a volar locking plate for fixation in combination with bone augmentation seems to provide better fixation, in terms of biomechanics, and seems to be a reasonable treatment [13]. However, the 2009 American Academy of Orthopaedic Surgeons (AAOS) guidelines could not recommend for or against the use of supplemental bone grafts or substitutes when using locking plates [4].

Currently, surgical treatment and volar locking plate fixation has become prominent, and it provides particularly good fixation for osteoporotic and comminuted bone fractures [6–9]. However, whether combination with bone augmentation is necessary to provide better support for fractures remains questionable; our review of the literature revealed that research in this regard is lacking [4, 14]. We hypothesized that surgical fixation of distal radius fractures using a volar locking plate with intraoperative bone augmentation could effectively prevent metaphyseal collapse. The purpose of this study was to verify this hypothesis and observe the effect of using a volar locking plate for internal fixation with and without bone augmentation on postoperative radiographic outcomes by comparing X-ray images immediately and at 6 months after surgery.

## Methods

### Patients and ethical considerations

In this retrospective cohort study, we examined the X-ray images of patients undergoing surgery for distal radius fracture at the Department of Orthopedics of Taichung Veterans General Hospital between January 1, 2014, and December 31, 2016. Patients were included if (1) they were aged > 18 years, (2) they had experienced a distal radius fracture, (3) they had undergone open surgery with internal fixation, (4) their surgery included the use of the DePuy Variable Angle LCP Two-Column Volar Distal Radius Plate 2.4 mm® (DePuy Synthes, Oberdorf, Switzerland), (5) they underwent surgery within 2 weeks of fracture, and (6) they were followed up postoperatively for at least 6 months. Patients were excluded from the study if: (1) they underwent re-fracture or revision surgery, (2) they had an associated ipsilateral ulnar shaft fracture, (3) their surgery was performed without using the plate mentioned above, (4) no locking screw was present on the main combi-hole of the plate, (5) they were followed up for less than 6 months postoperatively, and (6) their treatment included the use of an additional external fixation instrument. A total of 391 X-ray images of operated fractures were reviewed, and 105 fractures met the inclusion criteria.

### Measurements

We recorded background characteristics (age, sex, and ethnicity), fracture type (AO classification) [15], use of bone augmentation, associated injuries, radiographic outcomes, and complications (including infections, tendinopathies, neuropathies, and pseudoarthrosis).

**Fig. 1:**
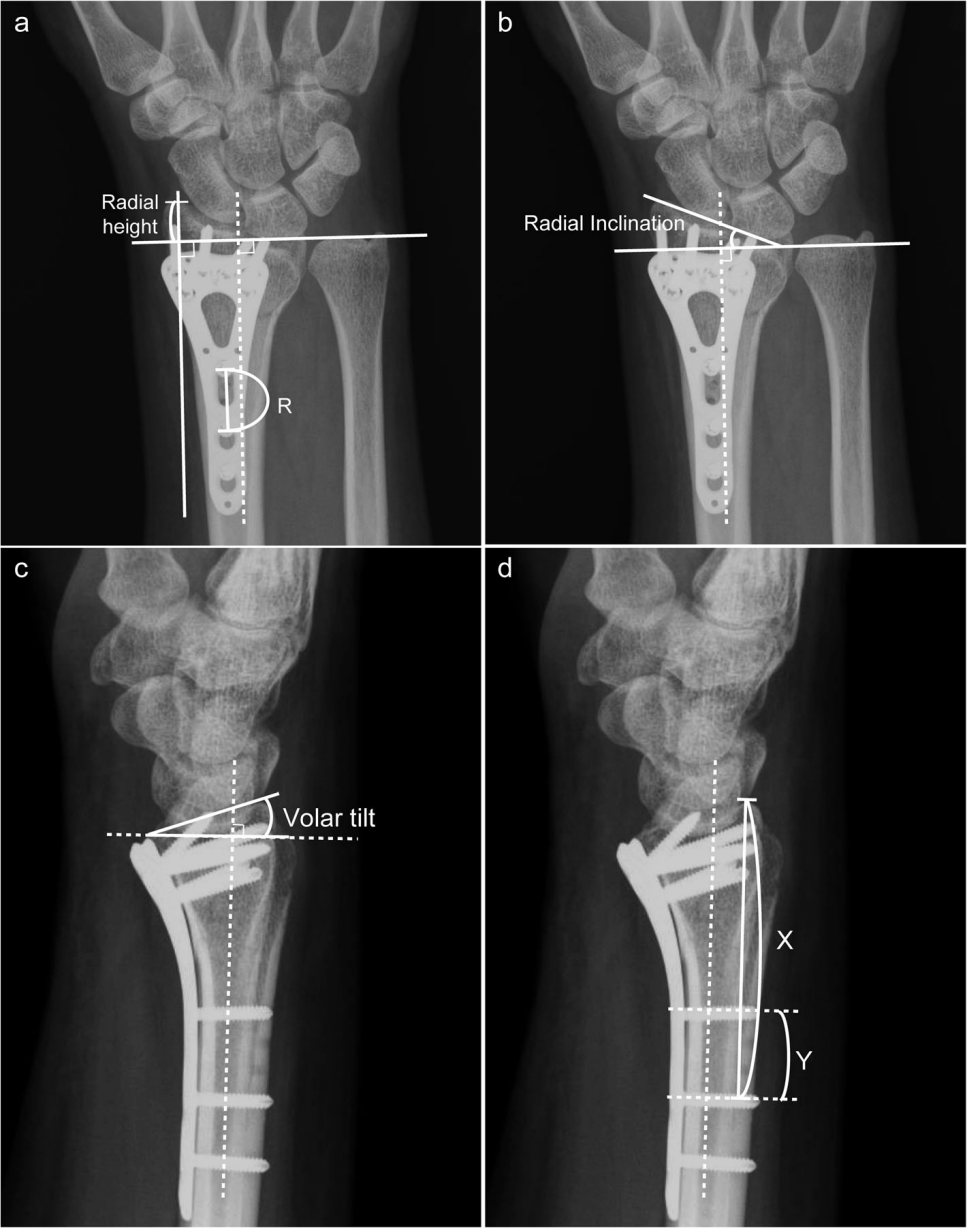
Radiographic measurements. **a** Radial height measurement. R is the distance from the distal 1st locking hole to the distal 2nd locking hole in the AP view, which should be fixed in each radiograph, so that that the Radial height/R ratio after correction is shown to minimize statistical error. **b** Radial inclination measurement is as shown in the figure. **c** Volar tilt measurement is as shown in the figure. **d** The status of dorsal collapse. X is the distance from the tip of the dorsal cortex to the 2nd distal locking screw in the lateral view. Y is the distance from the distal 1st to the distal 2nd locking screw in the lateral view. Y is a fixed value in each image, so that the ratio X/Y is shown to minimize statistical error

Using the postsurgical X-ray images obtained immediately and at 6 months after surgery, the following radiographic parameters were measured: radial height (Fig. 1a), radial inclination (Fig. 1b), volar tilting (Fig. 1c), and the status of dorsal collapse (Fig. 1d); the changes in each parameter were compared. The status of dorsal collapse was measured as follows: X was defined as the distance of the tip of the dorsal cortex to the 2nd distal locking screw; then, the change in X from immediately after surgery to 6 months after surgery was measured using the following equation:

$$ \Delta  \mathrm{X}={\mathrm{X}}_{6\mathrm{mo}}-{\mathrm{X}}_{\mathrm{postop}}. $$

**Fig. 2:**
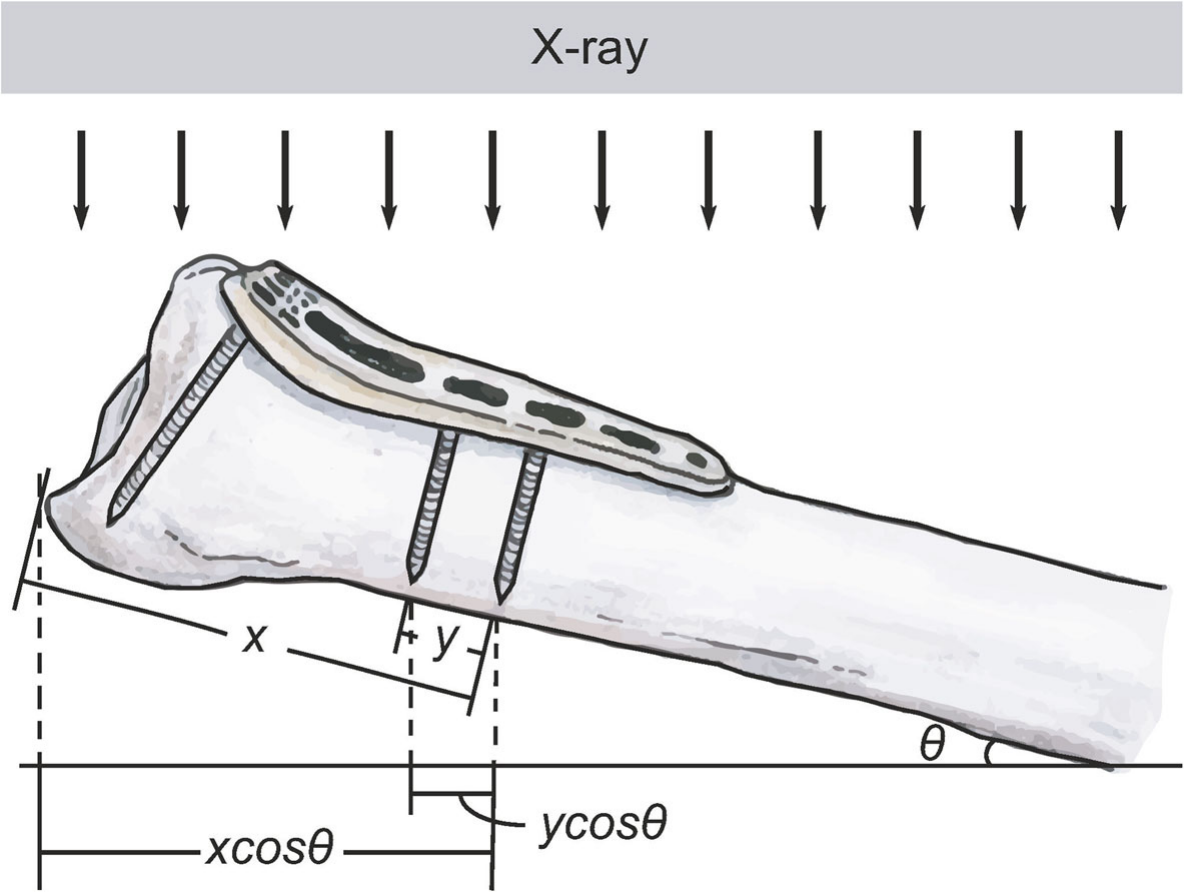
Correction for X-ray imaging angle. Because differences in the angle, θ, during each acquisition may cause errors, statistics are expressed as the ratio X cos θ/Y cos θ (= X/Y) for correction Cos, cosine.

Due to measurement errors in X-ray images caused by imaging angle or magnification, we made corrections during statistical analysis. Y was defined as the distance from the distal 1st to the distal 2nd locking screw. Y should be fixed between the images immediately and at 6 months after surgery. During each acquisition, X increases or decreases in proportion to Y. Therefore, statistical analysis using the ratio X/Y can eliminate magnification error. Furthermore, X and Y changed when the angle changes during acquisition. When Y becomes Y cos θ, X also becomes X cos θ. The ratio of X cos θ/Y cos θ could be used as an offset to achieve a corrective effect (Fig. 2). Therefore, in the statistical analysis, the status of dorsal collapse is shown as (Fig. 1d):

$$ {\mathrm{X}}_{6\mathrm{mo}}/{\mathrm{Y}}_{6\mathrm{mo}}-{\mathrm{X}}_{\mathrm{postop}}/{\mathrm{Y}}_{\mathrm{postop}} $$

**Fig. 3:**
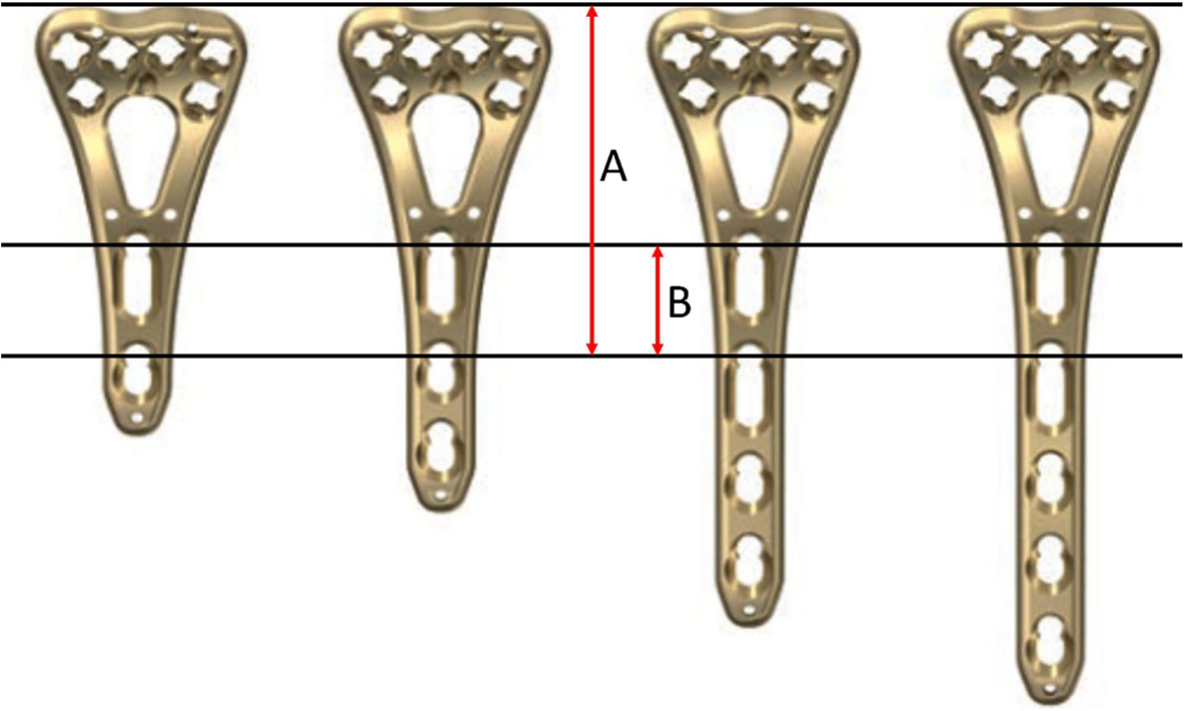
Comparison of plates of different lengths. The distance from the most distal end of the plate to the distal 2nd locking screw (**a**) is the same as the distance from the distal 1st to the distal 2nd screw (**b**), regardless of the length of the plate

In addition, the distance from the most distal end of the plate to the distal 2nd locking screw and the distance from the distal 1st to the distal 2nd screw of the DePuy Variable Angle LCP Two-Column Volar Distal Radius Plate 2.4 mm® steel plate used during the operation are the same regardless of the number of combi-holes; thus, even plates of different lengths can be compared with each other (Fig. 3).

Similarly, we made corrections for the radial height statistics. R was defined as the distance from the distal 1st locking hole to the distal 2nd locking hole on the anteroposterior (AP) view. The change in radial height (RH) from immediately after surgery to a 6-month follow-up is shown by (Fig. 1a):

$$ {\mathrm{R}\mathrm{H}}_{6\mathrm{mo}}/{\mathrm{R}}_{6\mathrm{mo}}-{\mathrm{R}\mathrm{H}}_{\mathrm{postop}}/{\mathrm{R}}_{\mathrm{postop}} $$

By evaluating the postsurgical X-ray images (immediate and after 6 months), we determined the status of fracture healing based on the radius union scoring system (RUSS); a RUSS score ≥ 6 represents healing [16].

### Surgery

Patients were admitted to the outpatient or emergency department and received a complete examination before surgery. Surgery was performed by all experienced attending surgeons working in the Department of Orthopedics of our hospital. Under general anesthesia, a tourniquet was inflated, and a modified Henry approach was used for entry. After correct reduction, we performed fixation using the DePuy Variable Angle LCP Two-Column Volar Distal Radius Plate 2.4 mm. The decision regarding bone augmentation was based on the judgment of each surgeon and conditions at the time of the operation. A bone substitute (“Purzer®” SinboneHT Bone Replacement; Taoyuan City, Taiwan, ROC) was used for all bone augmentation procedures by inserting it into the metaphyseal bone defect through the same volar incision, with no more than 5 mL per wrist. Finally, the C-arm examination was used to confirm the position of the plate and the condition of the fracture reduction to ensure that supination and pronation were feasible. After the pronator quadratus muscle was repaired as much as possible, the wounds were sequentially closed. The postoperative rehabilitation protocol was started based on an early active rehabilitation program [17], and a gentle active range of motion was started on postoperative days 3–5. Sutures were removed on postoperative days 10–14, and the wrist/forearm passive range of motion and strengthening exercises were started. Isotonic strengthening was started 4 weeks after surgery, and heavy putty strengthening was started at 6 weeks after surgery. Patients were evaluated at 3–5 days, 2 weeks, 1 month, 2 months, 3 months, and 6 months after surgery. Postoperative X-ray images were acquired immediately after surgery in the recovery room and at 1-month, 3-month, and 6-month follow-ups.

### Statistical analyses

To confirm the comparability of the two study arms, the Mann-Whitney U test was used for continuous variables (age) and the chi-squared statistic was used for normal variables (sex, injured side, and fracture type). The Mann-Whitney U test was also used to determine differences in radiographic parameters between the non-augmentation and augmentation groups immediately after surgery and at the 6-month follow-up. Furthermore, the Wilcoxon signed*-*rank test was used to determine differences in radiographic parameters between the non-augmentation and augmentation groups immediately after surgery and at 6 months postoperatively. A value of *p* < 0.05 was considered statistically significant. All statistical analyses were performed using SPSS Statistics version 24 (SPSS Inc., Chicago, Illinois, USA).

## Results

Table 1 summarizes the characteristics of patients in each group. Of the 105 patients, 60 were women (57.1%) and 45 were men (42.9%). The average age was 50.8 years (range: 19–83 years). Of the affected limbs, 46 were the right hand (43.8%) and 59 were the left hand (56.2%). According to the AO classification [15], 21 cases were classified as AO-type 23-A2, 30 as type A3, 1 as type B1, 2 as type B2, 6 as type B3, 21 as type C1, 16 as type C2, and 8 as type C3, of which 54 (51.4%) were considered as comminuted types (A3, C2, C3).

**Table 1:** Patient characteristics of the groups

	GP1 (***n*** = 88)		GP2 (***n*** = 17)		***p-***value
Age, years	52.50	(33.50, 63.00)	63.00	(51.50, 69.50)	0.011*
Sex					0.043*
F	46	(52.3%)	14	(82.4%)	
M	42	(47.7%)	3	(17.6%)	
Fragmentation					< 0.001**
0	50	(56.8%)	1	(5.9%)	
1	38	(43.2%)	16	(94.1%)	
R/L					0.574
R	37	(42.0%)	9	(52.9%)	
L	51	(58.0%)	8	(47.1%)	
Complications	9	(10.2%)	2	(11.8%)	> 0.99

Mann-Whitney U test, median (interquartile range). Chi-squared test, *p < 0.05, **p < 0.01

GP1, non-bone augmentation group; GP2, bone augmentation group

In all patients, internal fixation was performed using the DePuy Variable Angle LCP Two-Column Volar Distal Radius Plate 2.4 mm®. In 88 cases (83.8%), only the volar locking plate was used; these patients were designated as group 1 (GP1). In the other 17 cases (16.2%), a plate was used with bone augmentation; these patients were designated as group 2 (GP2).

We compared the differences in background characteristics between GP1 and GP2 and found that the age (*p* = 0.011), proportion of women (*p* = 0.043), and frequency of comminuted fractures (*p* < 0.001) were significantly higher in GP2; there was no significant difference in the side of injury between both groups (Table 2). There were no significant differences in radiographic outcomes between the two groups both immediately after surgery and at the 6-month follow-up (Table 3).

**Table 2:** Comparison of characteristics among patients with comminuted fractures

	GP1 (*n* = 38)		GP2 (*n* = 16)		*p-*value
Age, years	55.50	(43.00, 64.50)	63.00	(51.50, 69.50)	0.070
Sex					0.145
F	22	(57.9%)	14	(82.4%)	
M	16	(42.1%)	3	(17.6%)	
R/L					> 0.99
R	20	(52.6%)	9	(52.9%)	
L	18	(47.4%)	8	(47.1%)	

Mann-Whitney U test, median (interquartile range). Chi-squared test, *p < 0.05, **p < 0.01

GP1, non-bone augmentation group; GP2, bone augmentation group

**Table 3:** Differences in radiographic parameters immediately after surgery and at 6-month follow-up

	Non-augmentation group, GP1 (*n* = 88)					Augmentation group, GP2 (*n* = 17)					*p-*value†	*p-*value‡
	After surgery		At 6 months		*p-*value	After surgery		At 6 months		*p-*value		
For all patients with distal radius fracture												
*X/Y	3.56	(3.42, 3.74)	3.46	(3.34, 3.61)	< 0.001**	3.54	(3.36, 3.61)	3.42	(3.30, 3.53)	0.001**	0.399	0.289
Volar tilting	11.00	(9.00, 13.00)	11.00	(8.00, 13.00)	0.366	11.00	(7.00, 12.50)	10.00	(7.00, 13.00)	0.749	0.240	0.281
Radial inclination	22.00	(19.00, 24.00)	22.00	(20.00, 24.00)	0.171	22.00	(19.0, 24.00)	21.00	(19.50, 22.50)	0.233	0.730	0.278
Radial height/R^a^	0.57	(0.44, 0.66)	0.54	(0.39, 0.64)	< 0.001**	0.52	(0.43, 0.57)	0.45	(0.37, 0.57)	0.039*	0.171	0.196
For patients with comminuted fractures												
GP1, (*n* = 38)						**GP2 (*n*** **= 16)**						
X/Y	3.60	(3.48, 3.75)	3.54	(3.38, 3.64)	< 0.001**	3.52	(3.35, 3.61)	3.42	(3.29, 3.54)	0.001**	0.094	0.099
Volar tilting	11.00	(8.75, 13.00)	10.50	(8.00, 12.00)	0.098	11.00	(7.25, 12.75)	10.00	(7.00, 13.00)	0.497	0.402	0.621
Radial inclination	22.00	(19.00, 24.25)	22.00	(20.00, 24.25)	0.697	21.50	(19.00, 23.00)	21.00	(19.25, 22.00)	0.431	0.458	0.282
Radial height/R	0.59	(0.43, 0.65)	0.54	(0.39, 0.65)	0.002**	0.52	(0.46, 0.57)	0.46	(0.41, 0.57)	0.063	0.215	0.384

Mann-Whitney U test, median (interquartile range). Wilcoxon signed rank, *p < 0.05, **p < 0.01

†After surgery: non-augmentation vs. augmentation

‡At 6 months: non-augmentation vs. augmentation

*X was defined as the distance of the tip of the dorsal cortex to the 2nd distal locking screw

Y was defined as the distance from the distal 1st to the distal 2nd locking screw, which should be fixed between the images immediately and at 6 months after surgery. The ratio X/Y for statistical analysis can eliminate magnification error

^a^ R was defined as the distance from the distal 1st locking hole to the distal 2nd locking hole on the anteroposterior (AP) view, which should be fixed between the images immediately and at 6 months after surgery

The ratio Radial Height/R for statistical analysis can eliminate magnification error

**Fig. 4:**
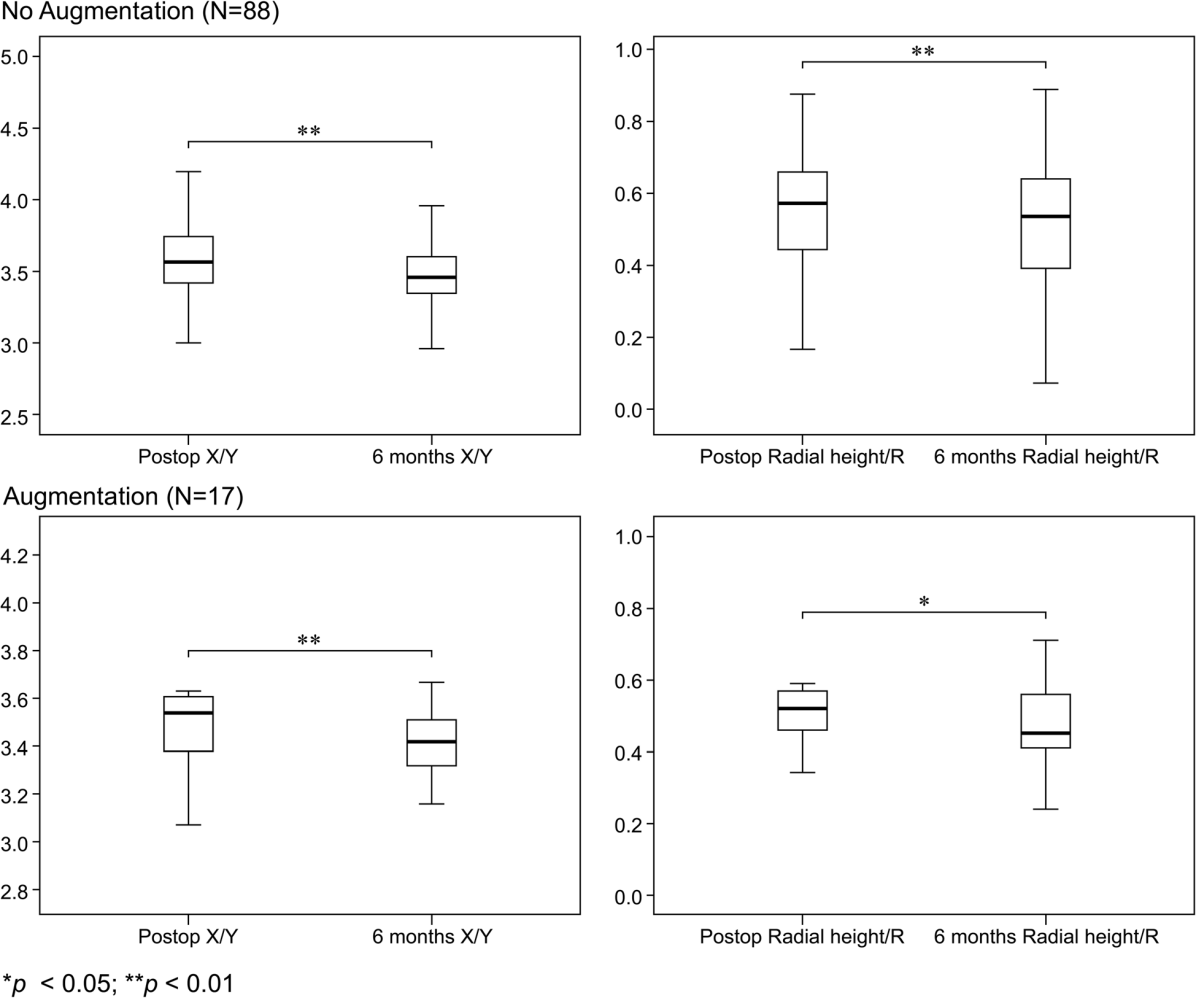
Six-month postoperative findings in the non-augmentation (GP1) and augmentation groups (GP2). Significant dorsal collapse and radial height shortening was noted at the 6-month follow-up in both the non-augmentation and augmentation groups

From the comparison of the postsurgical X-ray images taken immediately after surgery with those of the 6-month follow-up, we observed a significant difference in the change in dorsal collapse (∆X/Y) and radial height shortening in both groups, whereas no significant change in volar tilting or radial inclination was observed (Table 3, Fig. 4). However, there was no difference in the degree of dorsal collapse and radial height shortening between the two groups 6 months post-operation (Table 4). After 6 months, nearly all patients achieved union (103 patients, 98.1%). Only two patients (one in each group) had not met the criteria for the union, but both their RUSS scores were 5 points.

**Table 4:** Changes in radiographic parameters in the non-augmentation and augmentation groups

	Change				*p-*value
	Non-augmentation group, GP1 (***n*** = 88)		Augmentation group, GP2 (***n*** = 17)		
All patients with distal radius fractures					
X/Y	−0.08	(− 0.15, − 0.03)	− 0.10	(− 0.14, − 0.03)	0.715
Volar tilting	0.00	(−1.75, 1.00)	0.00	(− 1.50, 1.00)	0.874
Radial inclination	0.00	(−1.00, 2.00)	0.00	(−1.50, 0.50)	0.198
Radial height/R	−0.04	(−0.07, 0.01)	−0.05	(−0.09, − 0.01)	0.651
For patients with comminuted fracture types					
GP1 (*n* = 38)			**GP2 (*n*** **= 16)**		
X/Y	−0.10	(−0.17, − 0.04)	− 0.10	(− 0.13, − 0.02)	0.596
Volar tilting	0.00	(− 2.00, 1.00)	0.00	(−1.75, 1.00)	0.679
Radial inclination	0.00	(−1.00, 1.00)	0.00	(−1.00, 0.75)	0.458
Radial height/R	−0.03	(−0.09, 0.01)	−0.05	(−0.10, − 0.00)	0.583

Mann-Whitney U test, median (interquartile range). *p < 0.05, **p < 0.01

The majority of the patients (94 patients, 89.5%) did not suffer from the associated complications. Complications were noted in nine patients of GP1, and two patients of GP2. In GP1, six patients presented symptoms of carpal tunnel syndrome (three of them required implant removal after bone union), another two patients had acute surgical site infection (both resolved after oral antibiotics treatment), and the other patient suffered from De Quervain syndrome, which resolved after local injection treatment. In GP2, an extraosseous bone substitute was present in ten patients (58.8%), and two of them presented symptoms of carpal tunnel syndrome, and both of them required implant removal after the bone union. The extraosseous material had disappeared in six of the ten patients by 6 months. The overall complications were not significantly different between both groups. (*p* > 0.99) (Table 1).

Furthermore, of the 105 patients, 54 were diagnosed with comminuted fracture types (AO-type 23-A3, C2, C3). These patients were also divided into a non-bone augmentation group (GP1) and a bone augmentation group (GP2) with 38 and 16 patients, respectively. There were no significant differences in background characteristics, sex, or side of injury between the two groups (Table 2). There were no significant differences in radiographic outcomes between the two groups immediately after surgery and at 6-month follow-up (Table 3).

**Fig. 5:**
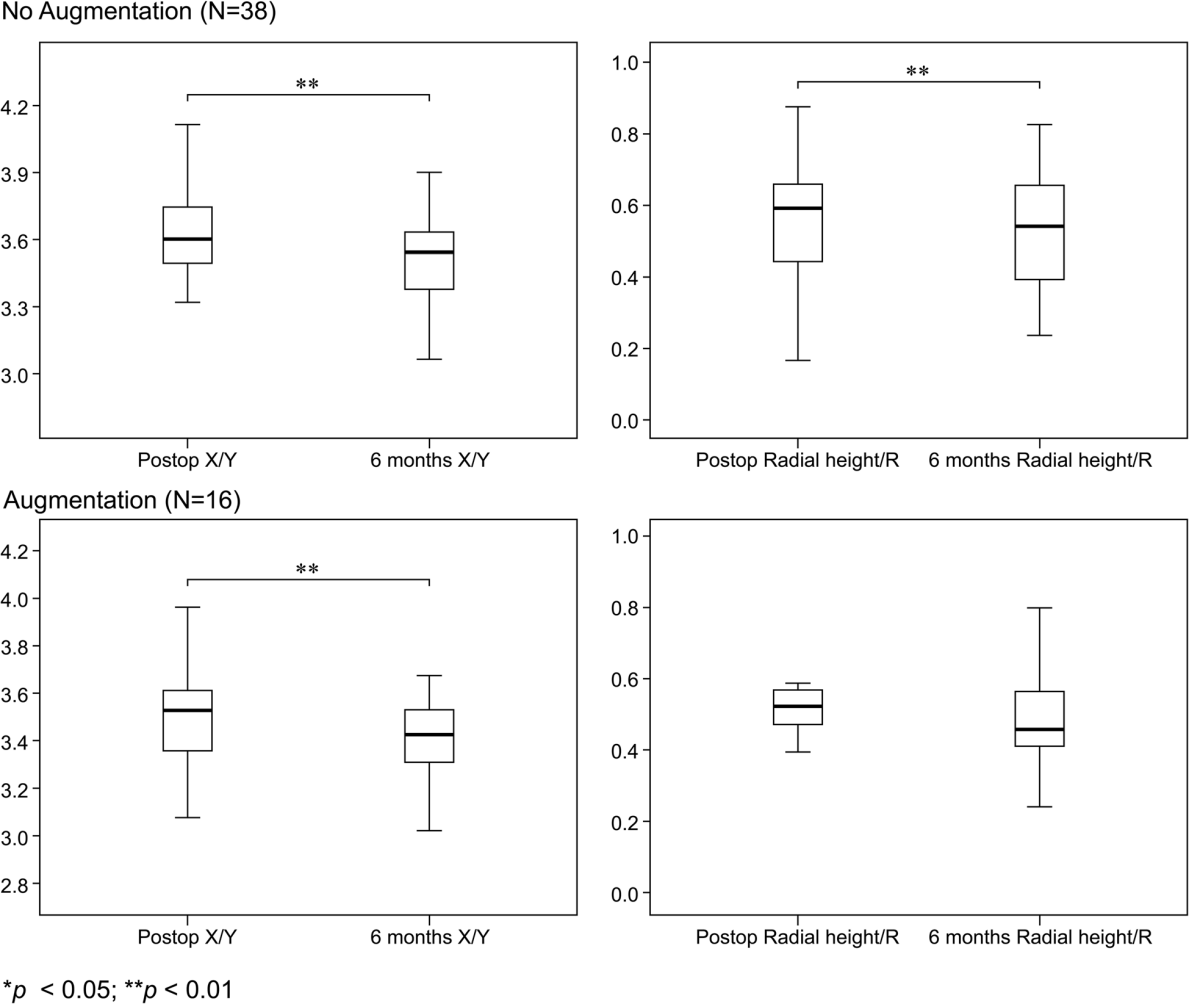
Six-month postoperative findings in patients with comminuted-type fractures. Significant dorsal collapse and a trend toward significance in radial height shortening were noted in both the non-augmentation and augmentation groups

The comparison of X-ray images immediately after surgery and at the 6-month follow-up revealed a difference in dorsal collapse (statistically significant) between both groups and a trend towards significance in radial height shortening (GP2); however, no significant change in volar tilting or radial inclination was detected (Table 3, Fig. 5). Furthermore, there was no significant difference in the degree of dorsal collapse and radial height shortening between groups (Table 4). Of the 54 patients, only one failed to meet the criteria for union and had a C2 fracture and required bone augmentation.

## Discussion

According to our comparisons of the postoperative X-ray images obtained immediately and at the 6-month follow-up, we found no significant change in volar tilting and radial inclination when using the volar locking plate for fixation, regardless of whether bone augmentation was performed. Most patients achieved bone union within 6 months and most of them (94/105 patients, 89.5%) did not have associated complications. Furthermore, a certain degree of dorsal collapse and radial height shortening was present, regardless of whether bone augmentation was performed; moreover, the degree of change in these parameters was not significantly improved by bone augmentation.

In addition, we performed statistical analysis including patients with comminuted fractures and reached similar conclusions. These data suggest that when the volar locking plate is used for fixation of distal radius fractures, combining it with intraoperative bone augmentation is not effective in preventing metaphyseal collapse. Although the complication rate was not significantly higher in bone augmentation group in our study, the additional cost and surgical time deem the technique less efficient. Instead, the fixation effect provided by the volar locking plate itself is adequate.

Since its development in 2000, the volar locking plate has become prominent for internal fixation. The volar approach is considered to have greater physiological advantages, as it can reduce irritation and damage to the extensor tendons and better preserve the dorsal metaphyseal blood supply. In addition, the reduced anatomical distance is achieved seamlessly because the volar side is usually less comminuted than the dorsal side. Furthermore, the physical characteristics of the locking compression plate design allow it to provide better fixation effects for osteoporotic bones and comminuted fractures. Its low-profile design can reduce irritation and damage to flexor tendons and the median nerve [6–9, 18, 19].

Although there are numerous advantages to the volar locking plate, complications can still arise. If possible, the plate should not be placed over the watershed line to avoid irritation and damage to the flexor tendons and the median nerve. Distal locking screws in an incorrect position or of an inappropriate length may also cause intra-articular screw penetration or extensor tendon irritation or rupture on the dorsal side [20, 21].

In fracture fixation, in addition to using the volar locking plate for internal fixation, the use of bone augmentation to fill metaphyseal bone defects has also been advocated, especially in osteoporotic and comminuted bone fractures, and it is believed that it can help prevent fracture collapse and promote bone healing. However, not many studies have demonstrated the indication, timing of use, and actual benefits of using bone augmentation [10–13].

Hegde et al. [10] used K-pin fixation combined with synthetic hydroxyapatite augmentation to treat 31 elderly patients with unstable distal radius fractures. After an average of 16 weeks of follow-up, they found no metaphyseal defects or collapse and satisfactory clinical outcomes among these patients.

In a cadaveric study, Högel et al. [13] used volar locking plates for fixation of AO-type 23-A3.3 fractures and divided patients into two groups: cement augmentation and non-augmentation groups. The results showed that cement augmentation significant increases the cycles and load until failure and provides better biomechanical properties.

However, a prospective randomized study [22] of 50 patients (24 patients underwent intraoperative combination with AlloMatrix bone graft and 26 did not) with unstable distal radius fractures treated with K-wire fixation found no difference in union rate and reduction parameters between the two groups after 1 year.

In a prospective study by Garcés-Zarzalejo et al. [14], 60 patients with unstable distal radius fractures were treated with the volar locking plate. All operations were performed without bone augmentation and all fractures achieved union without a significant loss of reduction after 1 year of follow-up.

As mentioned before, there is still controversy in the 2009 AAOS guidelines regarding the use of supplemental bone grafts or substitutes when using locking plates for the treatment of distal radius fractures. The decision depends on the experience of the surgeon and the preference of the patient [4].

In our study, we found that the volar tilting and radial inclination angles did not significantly change from the time of surgery to 6-month follow-up, regardless of whether bone augmentation was performed. From this, we inferred that the distal locking screws on the locking plate have functions similar to those of rafting screws, which support the subchondral bone, thereby maintaining volar tilting and radial inclination. Although the other parameters were slightly worse, this result is still generally consistent with that of other studies. That is, there is little effect on clinical outcomes, and most patients can achieve bone healing and satisfactory functional outcomes [14, 23–25].

Additionally, we attempted to correct the length (radial height, dorsal collapse) measurements in the anteroposterior and lateral views of the images in our study to minimize possible errors caused by each X-ray image acquisition, including those arising because of changes in the angle and magnification. However, it was not possible to correct the error in the angle (radial inclination and volar tilt) measurements. As far as we know, there are only a few studies that have corrected the measurements for this aspect of the images. Our study may be the first to attempt this correction during measurement.

Our study has some limitations. First, it was a retrospective study with a non-randomized design. Regarding measurements, we only made corrections to the length; we did not make corrections to the angle. Because the position and angle of wrist placement may cause errors during X-ray image acquisition, there is no absolute fixed angle that can be used as a parameter for correction. In addition, some errors may exist in the measurement of the images. Furthermore, the functional scores of the patients were not recorded in this study to judge clinical outcomes; only the radiographic outcomes were evaluated, but they could not be directly linked to clinical results. Although previous studies have reported that patients undergoing volar locking plate fixation for a distal radius fracture have some degree of deterioration of radiographic parameters on postoperative follow-up, they still had good clinical outcomes [14, 22–24]. Among our included patients, the number of patients in the bone augmentation group was relatively small, which may also bias our statistical results. However, this is because of the retrospective nature of the study. Based on the current literature, there is still a lack of research that demonstrates the absolute benefits of using bone augmentation. Currently, bone augmentation is performed based on the personal preferences of the surgeon and the intraoperative conditions. Future prospective studies should be randomized and include larger sample size and patients from multiple centers.

## Conclusion

The use of the volar locking plate is an effective and reliable treatment method for distal radius fractures. Even for comminuted fractures, no additional bone augmentation is needed if an anatomical reduction is achieved as much as possible and fixation is sufficient. Furthermore, nearly all patients in this study achieved union within 6 months of surgery with no loss of reduction.

## Data Availability

All data generated or analyzed during this study are available from the first author & corresponding author.

## Abbreviations

LCP: Locking compression plate
RH: Radial height
AAOS: American Academy of Orthopaedic Surgeons
RUSS: Radius union scoring system

## References

[1] Nellans KW, Kowalski E, Chung KC (2012). The epidemiology of distal radius fractures. Hand Clin.

[2] Huetteman HE, Zhong L, Chung KC (2018). Cost of surgical treatment for distal radius fractures and the implications of episode-based bundled payments. J Hand Surg.

[3] Koval K, Haidukewych GJ, Zirgibel BJ (2014). Controversies in the management of distal radius fractures. J Am Acad Orthop Surg.

[4] Lichtman DM, Bindra RR, Boyer MI, Putnam MD, Ring D, Slutsky DJ (2010). AAOS clinical practice guideline summary: treatment of distal radius fractures. J Am Acad Orthop Surg.

[5] Chung KC, Shauver MJ, Yin H, Kim HM, Baser O, Birkmeyer JD (2011). Variations in the use of internal fixation for distal radial fracture in the United States Medicare population. J Bone Joint Surg Am.

[6] Orbay JL, Fernandez DL (2004). Volar fixed-angle plate fixation for unstable distal radius fractures in the elderly patient. J Hand Surg [Am].

[7] Drobetz H, Bryant AL, Pokorny T, Spitaler R, Leixnering M, Jupiter JB (2006). Volar fixed-angle plating of distal radius extension fractures: influence of plate position on secondary loss of reduction--a biomechanic study in a cadaveric model. J Hand Surg.

[8] Osada D, Kamei S, Masuzaki K, Takai M, Kameda M, Tamai K (2008). Prospective study of distal radius fractures treated with a volar locking plate system. J Hand Surg.

[9] Fok MW, Klausmeyer MA, Fernandez DL, Orbay JL, Bergada AL (2013). Volar plate fixation of intra-articular distal radius fractures: a retrospective study. J Wrist Surg.

[10] Hedge C, Shetty V, Wasnik S, Ahammed I, Shetty V (2013). Use of bone graft substitute in the treatment for distal radius fractures in elderly. Eur J Orthop Surg Traumatol.

[11] Cassidy C, Jupiter JB, Cohen M, Delli-Santi M, Fennell C, Leinberry C (2003). Norian SRS cement compared with conventional fixation in distal radial fractures: a randomized study. J Bone Joint Surg Am.

[12] Handoll HHG, Watts AC. Bone grafts and bone substitutes for treating distal radial fractures in adults. Cochrane Database Syst Rev. 2008(2):CD006836. 10.1002/14651858.CD006836.pub2. 10.1002/14651858.CD006836.pub2 PMC8931728 18425972

[13] Hogel F, Mair S, Eberle S, Weninger P, von Oldenburg G, Augat P (2013). Distal radius fracture fixation with volar locking plates and additional bone augmentation in osteoporotic bone: a biomechanical study in a cadaveric model. Arch Orthop Trauma Surg.

[14] Garcés-Zarzalejo C, Sánchez-Crespo MR, Peñas-Díaz F, Ayala-Gutierrez H, Gimenez-Rico JS, Alfonso-Fernandez A (2015). Distal radius fractures: Should we use supplemental bone grafts or substitutes in cases of severe osteoporotic or comminution? (Article in English, Spanish). Rev Esp Cir Ortop Traumatol.

[15] Marsh JL, Slongo TF, Agel J, Broderick JS, Creevey W, DeCoster TA (2007). Fracture and dislocation classification compendium-2007: Orthopaedic trauma association classification, database and outcomes committee. J Orthop Trauma.

[16] Patel SP, Anthony SG, Zurakowski D, Didolkar MM, Kim PS, Wu JS (2014). Radiographic scoring system to evaluate Union of Distal Radius Fractures. J Hand Surg [Am].

[17] Brehmer JL, Husband JB (2014). Accelerated rehabilitation compared with a standard protocol after distal radial fractures treated with volar open reduction and internal fixation. J Bone Joint Surg Am.

[18] Orbay JL (2000). The treatment of unstable distal radius fractures with volar fixation. Hand Surg.

[19] Orbay JL, Touhami A (2006). Current concepts in volar fixed-angle fixation of unstable distal radius fractures. Clin Orthop Relat Res.

[20] Tanaka Y, Aoki M, Izumi T, Fujimiya M, Yamashita T, Imai T (2011). Effect of distal radius volar plate position on contact pressure between the flexor pollicis longus tendon and the distal plate edge. J Hand Surg [Am].

[21] Soong M, Earp BE, Bishop G, Leung A, Blazar P (2011). Volar locking plate implant prominence and flexor tendon rupture. J Bone Joint Surg Am.

[22] D'Agostino P, Barbier O (2013). An investigation of the effect of AlloMatrix bone graft in distal radial fracture: a prospective randomised controlled clinical trial. Bone Joint J.

[23] Kotian P, Mudiganty S, Annappa R, Austine J (2017). Radiological Outcomes of Distal Radius Fractures Managed with 2.7mm Volar Locking Plate Fixation-A Retrospective Analysis. J Clin Diagn Res.

[24] Vosbikian MM, Ketonis C, Huang R, Costanzo JA, Ilyas AM (2017). Reduction loss after distal radius fracture fixation with locked volar plates. J Surg Orthop Adv.

[25] Martinez-Mendez D, Lizaur-Utrilla A, de Juan-Herrero J (2018). Prospective study of comminuted articular distal radius fractures stabilized by volar plating in the elderly. Int Orthop.

